# Tissue and plasma levels of galectins in patients with high grade serous ovarian carcinoma as new predictive biomarkers

**DOI:** 10.1038/s41598-017-13802-5

**Published:** 2017-10-16

**Authors:** Marilyne Labrie, Lorenna Oliveira Fernandes De Araujo, Laudine Communal, Anne-Marie Mes-Masson, Yves St-Pierre

**Affiliations:** 1 INRS-Institut Armand-Frappier, Laval, Québec Canada; 2 Centre de recherche du Centre hospitalier de l’Université de Montréal (CRCHUM), Montréal, Canada; 3 Institut du cancer de Montréal, Montréal, Canada; 4 Department of Medicine, Université de Montréal, Montréal, Canada

## Abstract

Galectins are moving closer to center stage in detecting glycosylation aberration in cancer cells. Here, we have investigated the expression of galectins in ovarian cancer (OC) and examined their potential as biomarkers in tissues and blood plasma samples of high grade serous ovarian carcinoma (HGSC) patients. In tissues, we found that increased protein expression of stromal gal-1 and epithelial gal-8/9 was associated with a poor response to treatment of HGSC patients. Gal-8/9 were both independent predictors of chemoresistance and overall survival (OS), respectively. This galectin signature increased the predictive value of the cancer antigen 125 (CA125) on 5-year disease-free survival (DFS), post-chemotherapy treatment and 5-year OS. In CA125^LOW^ patients, epithelial gal-9 was associated with a lower 5-year OS while stromal gal-1 and epithelial gal-8 were both associated with a lower 5-year DFS. Such negative predictive value of gal-8 and gal-9 was also found using plasma samples. In both cases, high plasma levels of gal-8 and gal-9 was associated with a lower OS and DFS. Overall, these data suggest that galectins may be promising biomarkers to identify subgroups of HGSC patients with poorer prognosis. Our study also contributes to better define the heterogeneity of the disease.

## Introduction

The poor survival rate of patients with epithelial OC (EOC) is largely due to the high grade serous ovarian carcinoma (HGSC) histological subtype and advanced stage at presentation. Approximately 20% of patients diagnosed with HGSC do not respond to chemotherapy, and for those who initially respond, emergence of drug resistance during subsequent cycles of chemotherapy is a common problem that complicates the clinical decision-making process, along with other intrinsic factors (heterogeneity of the tumor, for example)^1,2^. It is thus critical to develop reliable and easily measurable biomarkers that provide a statistical probability to respond to therapy^3^. At present, the most commonly clinically validated biomarker for monitoring disease progression and assessing response and relapse to treatment is the carbohydrate antigen 125 (CA125). Unfortunately, serum CA125 lacks specificity and sensitivity, as a single marker, for early EOC detection and prognosis^4^. Moreover, the rate of false negatives is relatively high, especially in the early stages of ovarian cancer^5,6^.

Given the influence of the immune stroma on cancer progression, the development of panels of immune biomarkers with sensitivity and specificity of detection is a promising avenue to yield robust predictive tools for prognosis and monitoring response to therapy in ovarian cancer. Immune cells and cancer cells closely interact at every step of disease progression. Such cross-talk has a profound impact on disease progression as it can both inhibit and enhance tumor growth. Because of their known ability to induce local immunosuppression and to confer cancer cells with resistance to apoptosis, members of the galectin family are emerging as a new class of actionable immune biomarkers in cancer^7,8^. Galectins are soluble and small molecular weight proteins that are often expressed at abnormally high levels in cancer cells and cells of the tumor microenvironment^9–11^. Together with C-type lectins and siglec, galectins are among the major families of lectins that detect changes in the cellular glycome occurring during tumor transformation and progression^12,13^. Inside cancer cells, they are well known to confer resistance to drug-apoptosis or to increase the invasive behavior of tumor cells^14–16^. Galectins, however, are best known for their alarmin-like role as danger signals that neutralize cancer killing immune cells and inducing local and systemic immunosuppression^12,17–19^. Such immunosuppressive activity represents a major obstacle to cancer treatment and slows down the pace of progress in cancer immunotherapy, a promising avenue for the treatment of aggressive ovarian cancers^1,20–22^. Galectin family members, 13 of them have been identified in humans, have thus recently emerged as key proteins in the landscape of oncogenic signatures across human cancers^23,24^. In prostate cancer, staining of tissue biopsies collected at different stages of the disease has shown that while gal-1 was upregulated in more advanced lesions, gal-3 and gal-9 expression gradually decreased as cancer evolves. In contrast, gal-8 was expressed at moderate levels at all stages^24–26^. The potential of galectin signatures as predictive biomarkers has also been established in aggressive subtypes of breast cancer and has shown to be clearly distinct from that observed in prostate cancer. For example, in patients with triple-negative breast cancer, expression of nuclear gal-8 in epithelial cancer cells predicts a better DFS, distant-disease-free survival (DDFS), and OS, in contrast to high expression of nuclear gal-1 which correlates with poor DDFS and OS^27^. These studies indicate that specific galectin expression signatures contribute to the phenotypic heterogeneity of cancer and can be used to segregate subsets of aggressive cancer into clinically meaningful subtypes. The value of galectins as predictive biomarkers in ovarian cancer, however, remains unknown. In the present work, we have used plasma samples and tissue microarrays (TMAs) constructed from tumors collected from patients with HGSC to identify galectin signatures that provide prognostic information on the clinical behavior of tumors under active therapy and the relationship of the signatures to CA125.

## Results

### Galectin expression in normal ovaries and fallopian tubes

**Figure 1:**
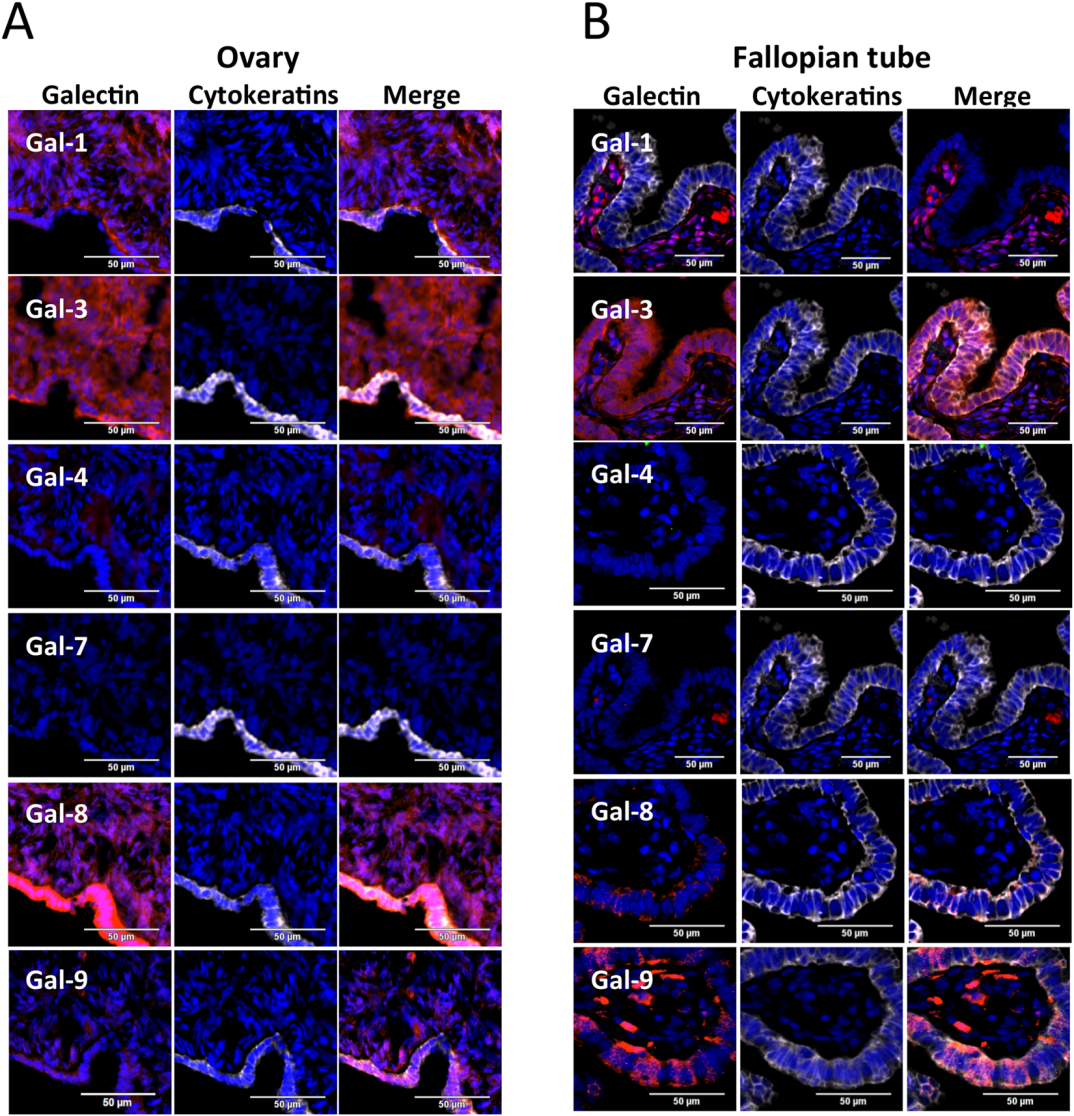
Galectins expression in normal ovarian and fallopian tube tissues. IF staining of gal-1, -3, -4, -7 -8 and -9 (Red) in normal (**A**) ovarian and (**B**) fallopian tube tissues. The characteristic cytosolic staining of cytokeratin staining (white) and DAPI were used to identify epithelial cells and nucleus, respectively. Cytokeratin negative tissue was considered as stroma. The merged images were used to determine localization of each galectins in both epithelial and stromal compartments. Bar represent 50 µm.

We first investigated gal-1, -3, -4, -7, -8, and -9 expression patterns by immunofluorescence (IF) using a tissue microarrays (TMAs) constructed from normal ovarian and fallopian tube tissues. In normal ovary, gal-1–3, -8, and -9 expression was evident in both the epithelial and stromal cells (Fig. 1). Cytosolic expression of gal-1, -3, -8, and -9 was clearly detectable in the epithelial lining, although the staining intensity for gal-9 was weaker. A similar pattern of expression was observed for stromal cells with the exception of gal-9, which showed strong nuclear staining in some cells. In Fallopian tubes, a strong expression of gal-3, -8, and -9 in terms of percentage of positive cells and intensity of staining was clearly evident in the epithelial lining while the stroma underneath the epithelial lining was strongly positive for gal-1 and contained some rare gal-7-positive cells. No detectable expression of gal-4 was found in fallopian tubes or the epithelial lining of the ovary, albeit weak yet clearly detectable expression was found in the stroma of the ovary.

### Expression of galectins in ovarian cancer subtypes

Our next series of experiments was carried out on a TMA containing a total of 63 patient samples of primary tumors representing several subtypes of ovarian carcinomas including serous, mucinous, endometrioid, and clear cell carcinomas (see Supplementary Table 1). Our results showed that a single tumor may express several galectins. An example of such a case can be found as supplementary Fig. 1A. This case is a mucinous ovarian cancer with a strong staining for gal-1, -3, -4, -8, and -9 while gal-7-positive cells were sparsely distributed. We also found that all subtypes harbor a relatively different galectin signature (see Supplementary Fig. 1B–E). While clear cell and endometrioid carcinomas often expressed gal-3, -7, -8, and -9, they rarely express gal-1 and gal-4. Gal-1 was also rarely expressed in mucinous carcinomas but was commonly found in serous OC, consistent with its well documented role in various aggressive subtypes of cancer^28,29^. In contrast, gal-4 was detected mostly in mucinous carcinomas and rarely found in serous, endometrioid or clear cell tumor tissues. Gal-3, -7, and -8 were commonly found in all histological subtypes. Expression of gal-9 was expressed in more than 80 percent of clear cell and mucinous samples but found in less than 40 percent of samples from patients with serous OC.

### Galectin expression in HGSC

**Figure 2:**
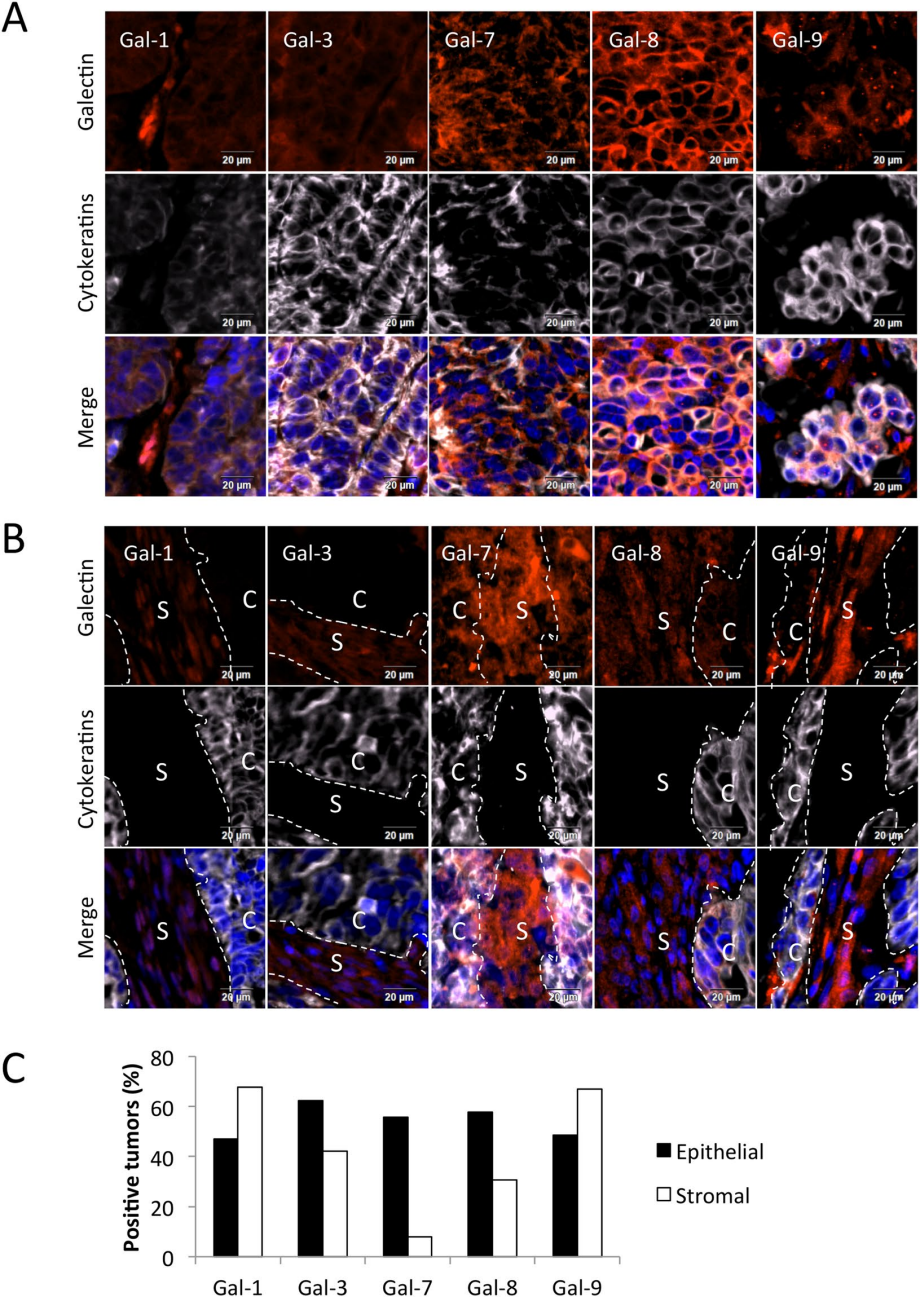
Galectins expression and tissular distribution in HGSC. Representative staining of gal-1, -3, -7, -8 and -9 (red fluorescence) in HGSC cancer cells (**A**) and peritumoral stromal cells (**B**). Cytokeratin staining (white fluorescence) and DAPI were used to identify epithelial cancer cells (compartment C) and nucleus, respectively. Cytokeratin negative tissue was considered as stroma (compartment S). Bar represent 20 µm. (**C**) Percentage of positive tumors expressing galectins in the cancer cells and the stroma of 209 HGSC specimens.

Our subsequent studies focused on TMAs constructed with 209 specimens of HGSC, the most aggressive subtype of EOC. The patients’ median age at diagnosis was 64.5 (range 36–89 years). By FIGO stage, 13 were stage I, 23 were stage II, 148 were stage III, and 25 were stage IV, consistent with the fact that most cases of HGSC are diagnosed at an advanced stage (see Supplementary Table 2). A particular attention was paid to galectin expression in stromal versus epithelial (cytokeratin-positive) cancer cells. Examples of such stromal vs. epithelial signatures observed for a given sample are shown in Fig. 2A,B. Our results showed that 45% to 65% of HGSC displayed epithelial expression of gal-1, -3, -7, -8 or -9 (Fig. 2C). The expression of gal-7, -8, and -9 was particularly strong in the cytosol. In epithelial cancer cells, gal-9 positive tumors often displayed cytosolic/perinuclear puncta (gal-9P), a feature that we also found in epithelial ovarian cancer cell lines (see Supplementary Fig. 2A–C). Staining with antibodies against LC3B and COX IV suggest that these aggregates were not associated to either autophagosomes or mitochondria respectively (see Supplementary Fig. 2D). In stroma, gal-1, -3, -8 and -9 were commonly found in cells surrounding the tumor. Stromal cells expressing gal-1 and -9 were found in 67% of cases while gal-3 and gal-8 were expressed in 42% and 31% of stromal cells, respectively. Gal-7 expression was rarely expressed in stromal cells (8% of cases) but commonly found in cancer cells (58% of cases).

### Prognostic values of galectins in HGSC

We found no significant association between epithelial gal-3, -7 and -9, as well as stromal gal-3 and -7 with any clinical parameters, including FIGO stage, recurrence of the disease and death (Table 1 and see Supplementary Table 3). Epithelial gal-1, however, was significantly (p = 0.016) associated with a higher CA125 values. There was also a strong tendency towards the association between epithelial gal-1 expression and a higher FIGO stage (p = 0.056) and the recurrence of the disease (p = 0.092). In univariate Cox analyses, epithelial gal-8 correlated with chemoresistance (HR, 1.757; 95% CI, 1.078–2.866, p = 0.024) while epithelial gal-9P correlated with a lower 5-year OS (HR, 1.537; 95% CI, 1.041–2.271, p = 0.031) (Table 2). The Cox proportional hazards model also showed that epithelial gal-8 was independently linked to chemoresistance (HR, 2.017; 95% CI, 1.082–3.750, p = 0.027) while epithelial gal-9P was independently linked to 5-year OS (HR, 1.734; 95% CI, 1.066–2.822, p = 0.027). In the case of stromal galectins, the only observed significant association we found was between stromal gal-1 and 5-year DFS (HR, 1.534; 95% CI, 1.071–2.199, p = 0.020). Multivariate analyses, however, showed that stromal gal-1 was not an independent prognostic factor (Table 2).

**Table 1:** Association between the patients characteristics and galectins expression.

	Gal-1 (stroma)		Gal-8 (epith)		Gal-9P (epith)		Gal-8 (plasma)		Gal-9 (plasma)	
	Low	High	Low	High	Low	High	Low	High	Low	High
Age^1^										
Mean	61	61	63	61	61	62	59	65	59	64
FIGO Stage^2^										
Low (I-II)	13 (41)	19 (59)	17 (50)	17 (50)	16 (47)	18 (53)	20 (69)	9 (31)	14 (50)	14 (50)
High (III-IV)	49 (31)	111 (69)	67 (41)	98 (59)	72 (46)	84 (54)	69 (58)	50 (42)	48 (41)	68 (59)
CA125^3^	*P = 0.125*									
Median	453.4	793	530.32	618	548	769.5	543	468	411	559
Residual disease^2^							*P = 0.001*			
No	10 (28)	26 (72)	18 (47)	20 (53)	20 (54)	17 (46)	26 (83)	5 (16)	17 (53)	15 (47)
Yes	42 (34)	80 (66)	58 (45)	70 (55)	53 (44)	67 (56)	45 (50)	45 (50)	33 (38)	53 (62)
5-yrs recurrence^2^	*P = 0.008*		*P = 0.065*				*P = 0.009*		*P = 0.015*	
No	18 (51)	17 (49)	21 (57)	16 (43)	18 (51)	17 (49)	26 (79)	7 (21)	20 (63)	12 (37)
Yes	41 (27)	112 (73)	62 (39)	96 (61)	70 (46)	81 (54)	59 (53)	52 (47)	41 (37)	69 (63)
5-yrs death^2^					*P = 0.109*		*P = 0.004*		*P = 0.028*	
No	31 (36)	54 (64)	36 (40)	53 (60)	45 (53)	40 (47)	55 (71)	22 (29)	37 (53)	33 (47)
Yes	31 (29)	76 (71)	48 (44)	62 (56)	43 (41)	62 (59)	34 (48)	37 (52)	28 (34)	49 (66)

^1^t-test,^2^Fisher exact test,^3^Kruskal-Wallis test.

**Table 2:** Univariate and multivariate Cox analysis.

Variables	Univariate analysis				Multivariate analysis			
	Hazard ratio	95% confidence interval			Hazard ratio	95% confidence interval		
		Lower	Upper	P-Value		Lower	Upper	P-Value
***5-years DFS***								
Age at diagnosis	1.005	0.989	1.021	0.541				
FIGO (Low-High)	3.376	2.036	5.598	**≤0.001**	2.208	1.192	4.090	**0.012**
Residual Disease	2.181	1.402	3.394	**≤0.001**	1.324	0.817	2.145	0.255
CA125 (Low-High)	1.770	1.249	2.509	**≤0.001**	1.634	1.105	2.418	**0.014**
Gal-1 (stroma)	1.534	1.071	2.199	**0.020**	1.396	0.920	2.117	0.117
Gal-8 (plasma)	1.706	1.177	2.474	**0.005**	1.489	1.001	2.214	**0.049**
Gal-9 (plasma)	1.621	1.1	2.388	**0.015**	1.795	1.140	2.825	**0.012**
***Chemoresistance***								
Age at diagnosis	0.995	0.972	1.017	0.638				
FIGO (Low-High)	5.240	2.516	10.912	**≤0.001**	3.360	1.353	8.350	**0.009**
Residual Disease	2.823	1.470	5.423	**0.002**	1.476	0.730	2.985	0.278
CA125 (Low-High)	2.701	1.588	4.593	**≤0.001**	2.630	1.390	4.979	**0.003**
Gal-8 (epith)	1.757	1.078	2.866	**0.024**	2.017	1.082	3.759	**0.027**
***5-years OS***								
Age at diagnosis	1.019	1	1.039	**0.046**	1.040	1.015	1.066	**0.002**
FIGO (Low-High)	3.138	1.589	6.198	**≤0.001**	2.530	1.040	6.155	**0.041**
Residual Disease	2.724	1.453	5.105	**0.002**	1.508	0.771	2.947	0.230
CA125 (Low-High)	1.862	1.193	2.905	**0.006**	1.933	1.159	3.225	**0.012**
Gal-8 (plasma)	2.110	1.327	3.356	**0.002**	1.724	1.034	2.875	**0.037**
Gal-9 (plasma)	1.693	1.046	2.743	**0.032**	1.799	1.008	3.211	**0.047**
Gal-9P (epith)	1.537	1.041	2.271	**0.031**	1.734	1.066	2.822	**0.027**

**Figure 3:**
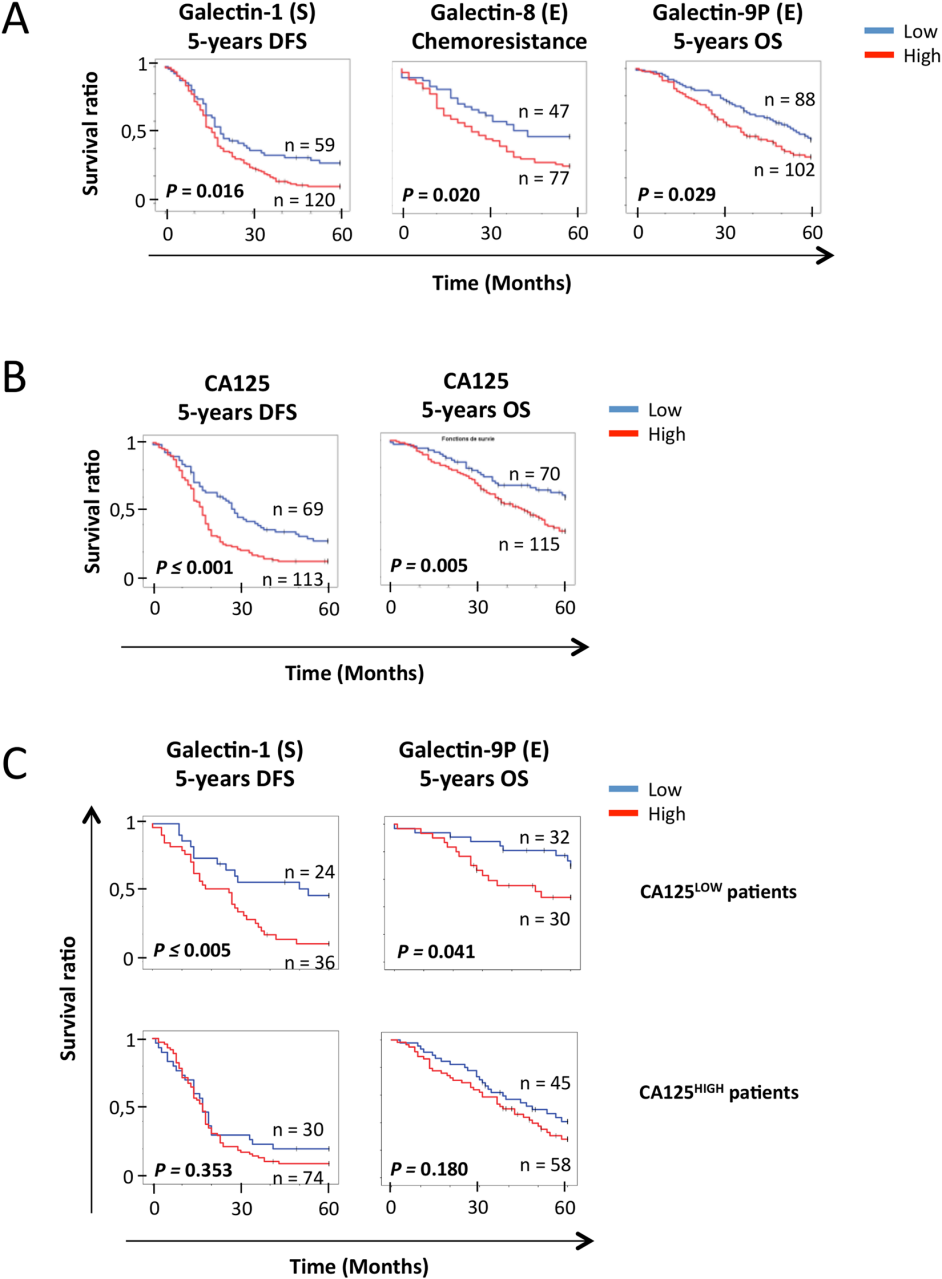
Galectins predictive value of OS, DFS and chemoresistance. (**A**) Kaplan-Meier curves of 5-years OS, 5-years DFS and chemoresistance according to stromal gal-1, epithelial gal-8 and gal-9P in HGSC samples. Blue bar: galectin low expression, red bar: galectin high expression. (**B**) Kaplan-Meier curves of 5-years OS and 5-years DFS according to the presence of circulating CA125. Blue bar: CA125^LOW^ samples, red bar: CA125^HIGH^ samples. (**C**) Kaplan-Meier curves of 5-years OS according to gal-9P presence and 5-years DFS according to stromal gal-1 presence in the tumors of CA125^HIGH^ or CA125^LOW^ patients. Blue bar: galectin low expression, red bar: galectin high expression. (S): Stroma; (E): Epithelium.

Patient survival curves, assigned by expression of each galectin to high and low groups, were next constructed via Kaplan-Meier method and were compared using the log-rank test. As shown in Fig. 3A, high expression of stromal gal-1 (p = 0.016), epithelial gal-8 (p = 0.020) and gal-9P (p = 0.029) was significantly predictive of poor DFS, chemoresistance, and 5-year OS, respectively. In a number of cases, we found an association between positive staining of a given galectin and 5-years OS, 5-years DFS or chemoresistance that fell just short of the traditional definition of statistical significance (see Supplementary Fig. 3). This was the case, for instance, for the association between 5-year OS and stromal gal-1 (p = 0.140) and gal-8 (p = 0.120) as well as epithelial gal-1 (p = 0.110) and gal-9 (p = 0.135).

We next investigated the predictive potential of galectins in the context of CA-125, the established biomarker for ovarian cancer. Consistent with previous findings, high plasma levels of CA-125 (CA125 ≥ 412 U/ml) was a risk factor for both 5-year DFS-survival and 5-year OS (Fig. 3B). While we found that galectins did not change the prognosis of patients with high plasma levels of CA-125, stromal gal-1 (p = 0.041) and epithelial gal-9 (p = 0.005) had a significant predictive value in patients with low plasma levels of CA125 (Fig. 3C). Taken together, our findings indicate that expression of gal-1 in stromal cells and gal-8 and gal-9 in epithelial cancer cells have a strong prognostic potential as molecular markers for HGSC.

### *Plasma levels of Galectin-8 and -9 as predictive biomarkers*

**Figure 4:**
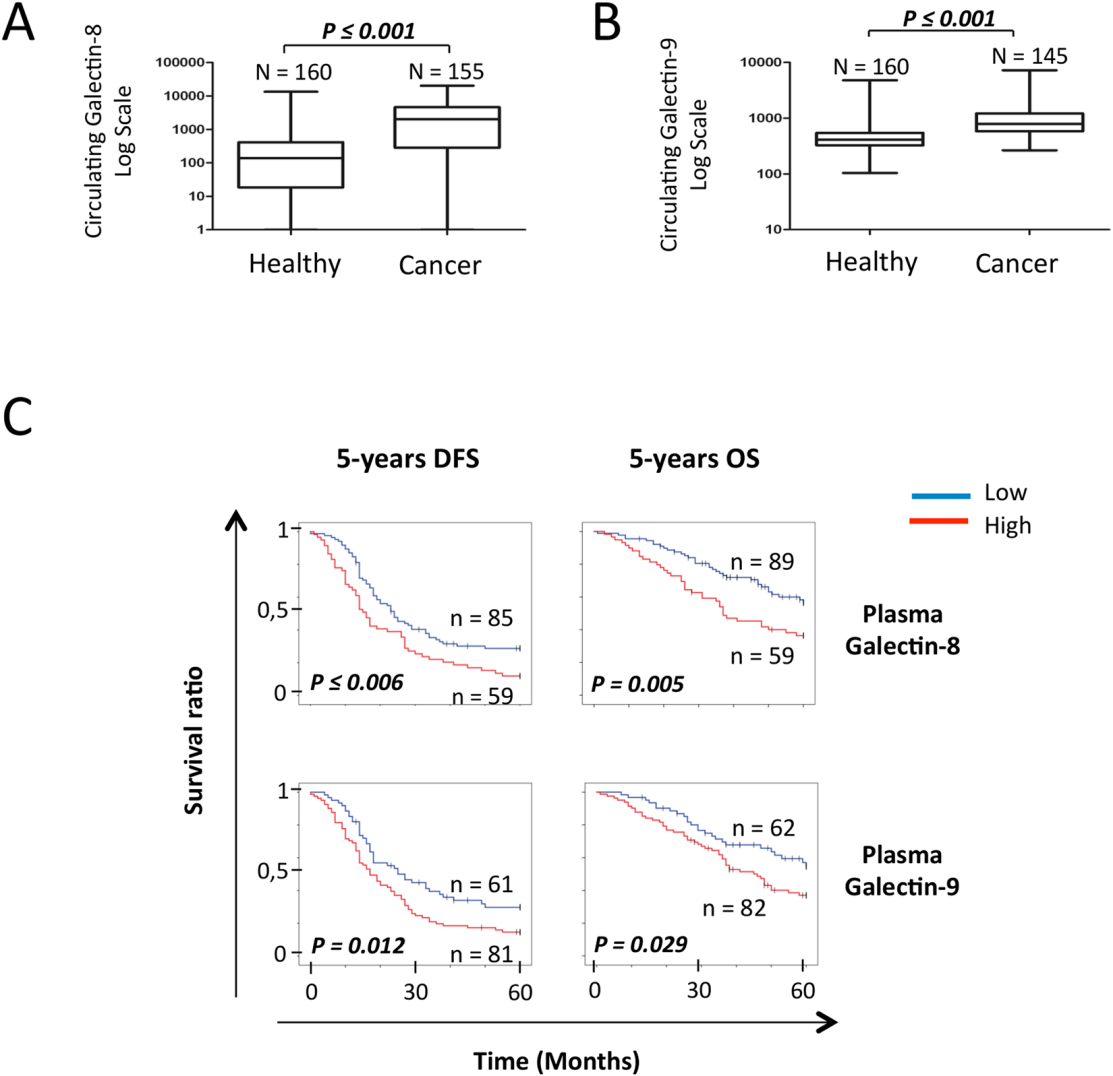
Predictive value of circulating galectins on the OS and DFS. (**A)** Gal-8 and (**B**) gal-9 concentration in the plasma of healthy donors and ovarian cancer patients. (**C**) Kaplan-Meier curves of the 5-years OS and 5-years DFS according to the presence of gal-8 and gal-9 in the plasma of HGSC patients. Blue bar: galectin negative samples, red bar: galectin positive samples.

**Figure 5:**
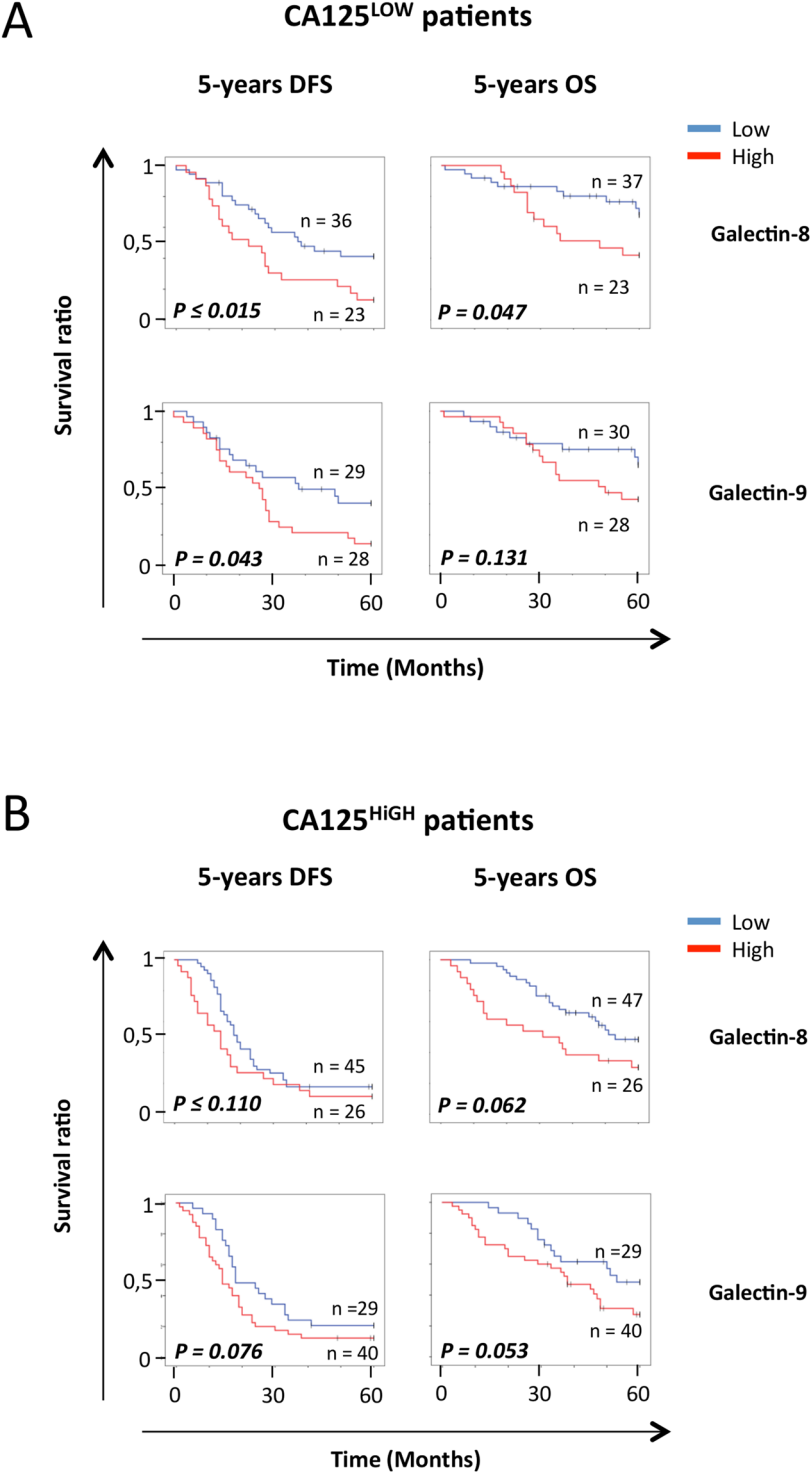
Predictive value of circulating galectin-8 and -9 according to CA-125 levels. Kaplan-Meier curves of 5-years OS and 5-years DFS according to the presence of circulating gal-8 and gal-9 in the plasma of (**A**) CA125^LOW^ or (**B**) CA125^HIGH^ patients. Blue bar: galectin negative samples, red bar: galectin positive samples.

**Figure 6:**
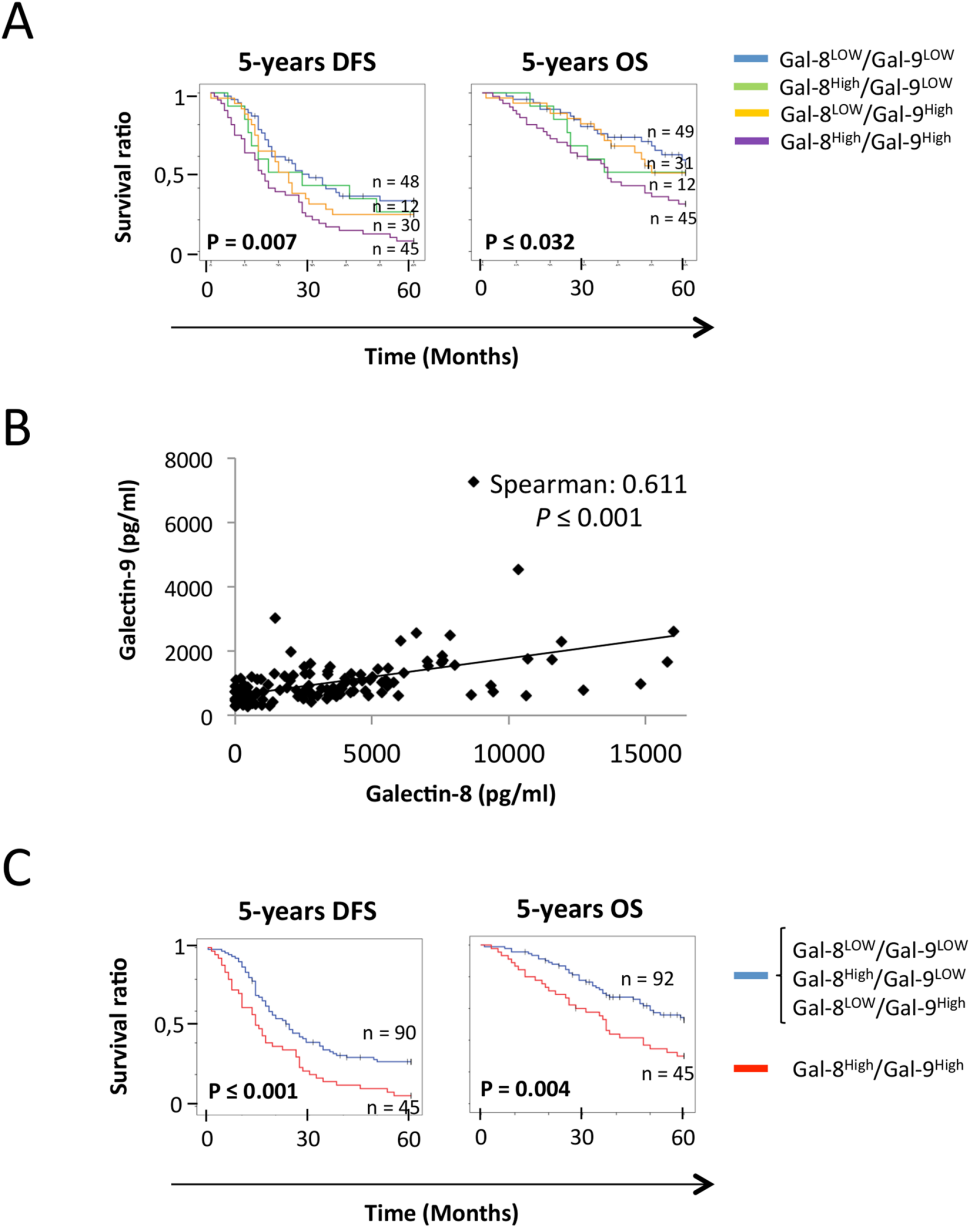
Combining gal-8 and gal-9 increases the predictive value for OS and HFS. (**A**) Kaplan-Meier curves of the 5-years OS and 5-years DFS according to the presence of either or both gal-8 and gal-9 in the plasma of HGSC patients. Blue bar: Gal-8^LOW^/Gal-9^LOW^, green bar: Gal-8^High^/Gal-9^LOW^, Yellow bar: Gal-8^LOW^/Gal-9^High^, Magenta bar: Gal-8^High^/Gal-9^HIGH^. (**B**) Correlation between the concentration of gal-8 and gal-9 in the plasma of HGSC patients. (**C**) Kaplan-Meier curves of the 5-years OS and 5-years DFS according to the presence of both gal-8 and gal-9 in the plasma of HGSC patients. Blue bar: Gal-8^LOW^/Gal-9^LOW^, Gal-8^High^/Gal-9^LOW^ or Gal-8^LOW^/Gal-9^High^ sample; Red bar: Gal-8^High^/Gal-9^HIGH^ samples. Patients with plasma gal-8 ≥ 2.7 ng/ml or plasma gal-9 ≥ 0.73 ng/ml were considered Gal-8^High^ and Gal-9^High^, respectively.

Although tissue staining represents a valuable tool to assess the heterogeneity of tumors and has strong potential for the development of biomarkers with high clinical sensitivity and specificity, ELISA-based measure of protein biomarkers remains the gold-standard in clinical setting. We have thus examined whether galectins are expressed at abnormally high levels in patients with HGSC and can be used as a predictive tool. Our preliminary results using a limited number of specimens (n = 35) showed no significant differences in plasma levels of gal-1 between healthy controls (n = 35, mean: 33.47 ± 79.20 ng/ml) and patients with HGSC (n = 30, mean: 21.79 ± 80.94 ng/ml) (see Supplementary Fig. 4A), consistent with a previous study showing that plasma levels of gal-1 had no predictive value in ovarian cancer^30^. In contrast, levels of gal-8 (n = 34, mean: 2.08 ± 2.86 ng/ml) and gal-9 (n = 30, mean: 1.15 ± 1.24 ng/ml) were significantly higher in HGSC patients as compared to healthy controls (n = 35; mean plasma levels of gal-8: 0.25 ± 1.70 ng/ml; mean plasma levels of gal-9: 0.50 ± 0.18 ng/ml) (see Supplementary Fig. 4B,C). For the remaining of our study, we thus focused on gal-8 and gal-9, for which there are no published reports on their predictive value in EOC. Overall, we have measured plasma levels of gal-8 and gal-9 in 160 specimens from healthy controls and from 155 and 145 specimens for gal-8 and gal-9, respectively. Our results showed that gal-8 (p ≤ 0.001) and gal-9 (p ≤ 0.001) plasma levels were significantly higher in patients with HGSC as compared to healthy controls (Fig. 4A,B). High plasma levels of gal-8 were associated with a lower 5-year DFS (p ≤ 0.006) and 5-year OS (p = 0.005) (Fig. 4C). A similar association was found between high plasma levels of gal-9 and a lower 5-year DFS (p = 0.012) and 5-year OS (p = 0.029). High plasma levels of gal-8 were predictive for 5-year DFS (p = 0.015) and 5-year OS (p = 0.047) in patients with low CA125 plasma levels (Fig. 5A). In patients with high CA125 plasma levels, a high plasma levels of gal-8 also showed a tendency of a lower 5-year OS (p = 0.062) (Fig. 5B). In the case of plasma gal-9, its level in patients that were CA125^LOW^ was only associated with 5-year DFS (p = 0.043), although associations with 5-year DFS (p = 0.076) and 5-year OS (p = 0.053) in patients that were CA125^HIGH^ showed a clear tendency to significance. Combining plasma levels of gal-8 and gal-9 had a significant effect on 5-year DFS (p = 0.007) and 5-year OS (p = 0.032) (Fig. 6A). Spearman correlation coefficient analysis showed that plasma levels of gal-8 did indeed correlate with those of gal-9 (r = 0.611 p ≤ 0.001) (Fig. 6B). Patients with both gal-8 and gal-9 high levels had significant lower 5-year DFS (p ≤ 0.001) and 5-year OS (p = 0.004) (Fig. 6C). Multivariate analysis further showed that plasma levels of gal-8 and gal-9 are both independent predictors of 5-year DFS (gal-8: HR, 1.489; 95% CI, 1.001–2.214, p = 0.049; gal-9: HR, 1.795; 95% CI, 1.140–2,285, p = 0.012) and 5-year OS (gal-8: HR, 1.724; 95% CI, 1.034–2.875, p = 0.037; gal-9: HR, 1.799; 95% CI, 1.008–3.211, p = 0.047) (Table 2).

## Discussion

The treatment of OC is severely complicated by high frequency of disease recurrence and the development of resistance to treatment. This problem is exacerbated by the absence of reliable biomarkers that can identify with high accuracy patients that would benefit. In search for new biomarkers for OC, we have studied expression of galectins in tissues and plasma of patients with HGSC samples. Our results revealed that OC are heterogeneous in their expression patterns of galectins. We have also identified a galectin signature that takes into account the expression of multiple galectins in both cancer tissues and plasma. More specifically, we found that galectin levels in plasma and expression in both cancer and peritumoral stromal cells can potentially be used to predict 5-year DFS, chemotherapy response and 5-year OS in HGSC patients and to potentiate the predictive value of CA125. If validated, galectin signatures may be very useful to help clinicians to identify patients with a high probability of resistance to chemotherapy, especially in patients who are CA125^LOW^.

Glycobiology is a promising avenue for the development of novel biomarkers that target altered glycosylation, a universal feature of cancer cells. Not surprisingly, there is increasing interest at examining the potential of galectins as predictive biomarkers. Such a strategy has recently been exploited to stratify cancer patients and to develop powerful predictors of cancer prognosis, recurrence risk, and metastasis^31–34^. Although most studies have focused on gal-1 and gal-3 as predictive biomarkers, recent studies have shown that multiple galectins are expressed at abnormally high levels in cancer. In breast cancer, analysis of the stromal signature showed that gal-1, -3, -9-positive stroma were preferentially found in triple-negative and HER2 subtypes^27^. Such expression of gal-1, -3, and -9 signature has also been reported in the stroma and cancer cells in the case of squamous cervical cancer^35^. This signature is somewhat similar to that we found here in HGSC. However, although gal-1 correlated with a poor prognosis in all cases, the expression pattern and prognostic value of gal-9 seem to differ according to the type of cancer. In squamous cervical cancer, expression of gal-9 is not abundant in either the tumor epithelium or the stromal cells and has been associated with a better survival^36^. This was clearly not the case for HGSC. An association between high levels of gal-9 with a reduced survival has also been reported in a recent study in melanoma^37^. Such differences might be explained by distinctive features of types of cancer. This is certainly the case for gal-7, which inhibits the development of colon cancer while increasing metastasis of lymphoma and breast cancer cells^38–40^. Alternatively, the dual role for a galectin in cancer cells can be attributed to distinct subcellular localization^27,41,42^. This has already been well documented for gal-3^43^ and more recently for nuclear gal-8^44^. Such heterogeneity of the subcellular localization of galectins and their distinct functions significantly complicates the interpretation of expression results obtained at the mRNA level and further emphasize the relevance of using immunohistochemical subcellular localization analyses to accurately measure the predictive potential of galectins and other proteins that are known to traffic from one cell type to the other. Our study has shown, however, that ELISA testing of galectins in the plasma of HGSOC patients is an equally promising avenue for the use of galectins as predictive biomarkers. The use of plasma biomarkers is not only less invasive and less costly, it represents a better way to assess or bypass spatial heterogeneity of tissue biopsies. This might explain why plasma levels of a given galectin do not always reflect the expression levels observed in primary tumors (see Supplementary Table 4). We indeed found that gal-8 and gal-9 plasma levels did not always correlate with expression levels in tissues. Yet, in both IF and ELISA, the predictive value of gal-8 and -9 was associated with a bad prognosis, indicating that both methods are adequate to obtain a closer follow up of HGSC patients’ condition and response to treatment using galectins as biomarkers. Although we observed the same association with epithelial gal-8 and chemoresistance at 6 month post-chemotherapy treatment (see Supplementary Fig. 3D), it is important to note that because of the limited number of patients used in this study, we considered chemoresistance as a relapse within 2-years post-chemotherapy treatment. Chemoresistance is often characterized as a relapse during the 6 months following the last chemotherapy treatment^45^, although longer periods of time have also been associated with increased chemoresistance^46,47^. Future studies using larger cohorts of patients will be needed to correlate expression of galectin signatures with a specific treatment and ideally a single agent will thus be needed to confirm our results using other criteria of chemoresistance. Another interesting venue will be to investigate whether plasma gal-8 and -9 expression levels can be used to monitor OC progression before, during and after treatment. Such a tool would be highly valuable in the prediction of the treatment efficacy and early recurrence detection.

Historically, galectins are best known for their role outside the cells following their secretion in the extracellular space via a non-classical pathway. Among their extracellular functions, galectins exert a profound immunosuppressive effect on immune cells. This has been well characterized for gal-1^48–51^. In all cases, higher gal-1 expression levels in either epithelial cancer cells, tumor microenvironment or in liquid biopsies correlate with a poor prognosis^24,52–57^. Our results showing that higher expression of gal-1 in stromal cells correlated with reduced 5-year OS is consistent with a recent study in EOC by Chen *et al*.^58^. In contrast to gal-1, however, research on the role of other less well known galectins, including gal-7, -8, and -9, outside the cells is just at its infancy but will likely expand at a rapid pace given the importance of glycosylation in the regulation of immune checkpoints. Yet, it is already clear that they do exert a regulatory function of immune cells and have strong potential as biomarkers. In the case of gal-9 in particular, its circulating levels has been investigated in patients with immune disorders or during infections^59–61^. These studies certainly provide impetus for future investigations on the correlation between plasma levels of gal-9 and immune status of HGSC. Because extensive splicing has been reported for gal-9, it will also be interesting to compare the prognostic value of each isoform in HGSC, as recently shown by Shulkens *et al*. in NSCLC^56^, and whether the isoforms have distinct cellular localization that correlate with their prognostic potential, as in the case of other galectins. This may explain why gal-9 displays a different intracellular distribution pattern in various cell lines (see Supplementary Fig. 2).

In summary, our study supports the idea that galectin signatures may be useful for the follow up of patients with ovarian cancer. We believe that such signatures could be used to distinguish between benign and malignant disease and to monitor response to therapy in women with ovarian cancer. Future work with independent cohorts of patients will be necessary to confirm the predictive value of this signature and to determine whether this signature can be incorporated in multiple biomarkers panels for risk stratification of patients with ovarian cancer.

## Materials and Methods

### Ethics statement

A written informed consent was obtained from all subjects providing tissue specimens. This study was conducted in accordance with guidelines and approval of the institutional ethical review boards of the Centre Hospitalier de l’Université de Montréal (CHUM) ethics committee.

### Tumor, plasma and normal tissue specimen

Normal tissue specimens, tumor and match plasma were obtained from patients treated at the CHUM from 1992 to 2012. All tumor samples were evaluated by a gynecologic-oncologic pathologist who assigned tumor grade and histopathological subtype according to the criteria established by the International Federation of Gynecology and Obstetrics. The disease stage was determined at time of surgery. Only tumors from patients that did not receive preoperative chemotherapeutic treatment were included in this study. The clinical data on disease-free interval were defined according to computed tomographic (CT) imaging, alone or combined with blood CA125 levels. Formalin-fixed paraffin-embedded material from each primary tumor and normal tissue samples was used to construct TMA. For IF, three different TMAs were used: the multi-subtype TMA, with 63 samples of various EOC subtypes (see Supplementary Table 1) and 8 normal ovarian tissue samples, the HGSC TMA with 209 samples of HGSC^62^ (see Supplementary Table 2) in duplicate and a TMA with a total of 14 normal fallopian tubes samples. For the ELISA experiments, plasma samples from a total of 160 healthy donors and 160 cancer patients were used. All plasma samples came from the same cohort of patients used to build the HGSC TMA.

### Immunofluroescence (IF)

Immunostaining reactions were performed using the BenchMark XT automated stainer (Ventana Medical System Inc., Tucson, AZ). Deparaffinized sections were incubated in cell conditioning 1 buffer (pH 8.0) (Ventana Medical System Inc.) for antigen retrieval and then stained for 60 min with the mouse anti-gal-1 (1:4000, Proteintech Group, IL, Cat. No 3G10D2), rabbit anti-gal-3 (1:2000, Abcam, MA, Cat. No. EP2775Y), goat anti-gal-4 (1:300, Santa Cruz, CA, Cat. No. T20), goat anti-gal-7 (1:100, R&D Systems,MN, Cat. No. AF1339), rabbit anti-gal-8 (1:50, Abcam, Cat. No. Ab183637)), rabbit anti-gal-9 (1:100, Abcam, Cat. No. Ab69630) and a mix of mouse anti-cytokeratin-7 (1:200, Thermo Scientific, Cat. No. OV-TL 12/30), anti-cytokeratin-18 (1:200 Santa Cruz, Cat. No. DC-10) and anti-cytokeratin-19 (1:200 Thermo Scientific, Cat. No. A53-B/A2.26). After washing three times in PBS, sections were incubated for 45 min with secondary antibodies consisting of a goat anti-mouse Alexa Fluor 750 (1:500, Life Technologies, ON, Canada), goat anti-mouse Cy5 (1:500 Life Technologies), donkey anti-goat-biotin (1:300, Jackson ImmunoResearch, PA), streptavidin Alexa Fluor 488 (1:500, Jackson ImmunoResearch), donkey anti-goat Alexa Fluor 488 (1:500, Life Technologies), goat anti-rabbit Cy5 (1:500, Life Technologies) and goat anti-rabbit Alexa Fluor 488 (1:500, Life Technologies). Nuclei were stained with 4’,6-diamidino-2-phenylindole (DAPI). The slides were scanned with a 20 × 0,75NA objective with a resolution of 0,3225 μm (VS110, Olympus, Center Valley, PA) and the intensity of the staining was quantified as mean fluorescent intensity (MFI), using VisiomorphTM software (Visiopharm, Denmark). For the stroma, the percentage of staining was scored from 0 to 3 according percentages of positive cells displaying the protein expression within a sample (0 = 0%; 1 ≤ 30%; 2 ≤ 60%; 3 ≥ 61%). The intensity of staining was also scored from 0 to 3, with a score of 0 representing no detectable staining and a score of 3 representing the strongest staining. Histological scores were calculated by multiplying both percentage and intensity scores. Histological scores higher than 3 were considered as high expression levels. The validation of antibodies specificity was assessed by western blot and IF using lysates of ovarian cancer cell lines and TMA of those respective cell lines.

### ELISA

The concentration of gal-1, gal-8 and gal-9 was measured in plasma of healthy donors (n = 160) and cancer patients (n = 160) (see Supplementary Fig. 3). For ELISA testing, plasma samples were diluted 3-fold for galectin-1 (R&D Systems) and galectin-8 (Ray Biotech), and 10-fold for galectin-9 (R&D Systems). All ELISAs were performed according to the manufacturer’s recommendations.

### EOC cell lines

The HGSC cell line OV-4453 was established from the ascites of a patient whereas TOV-1369TR and TOV-1369 (2) were established from a HGS ovarian tumor of a patient before (TOV-1369TR) and after (TOV-1369 (2)) chemotherapy treatment^63,64^. All cell lines were maintained in ovarian surface epithelium (OSE) medium (Wisent, QC, Canada) supplemented with 10% [v/v] fetal bovine serum.

### Immunocytochemistry

Cells were fixed in 3% (w/v) paraformaldehyde for 15 min, permeabilized in 0.1% (v\v) PBS/Triton X-100 for 5 min and blocked overnight at 4 °C in 1% (w/v) PBA. Rabbit anti-human gal-9 (1:100, Abcam), goat anti-human gal-9 (1:50, R&D Systems), rabbit anti-human LC3B (1:200, Sigma) and rabbit anti-human Cox IV (1:500, New England Biolabs, Ipswich, MA) primary antibodies were used. Secondary antibodies were a donkey anti-rabbit Alexa Fluor 647 (1:500, Life Technologies) and a donkey anti-goat Alexa Fluor 488 (1:500, Life Technologies). All antisera were diluted in 1% (w/v) PBA, and all washing steps were performed with PBS. Nuclei were stained with ProLong Gold Antifade Reagent with DAPI (Life Technologies). Cells were visualized under a Carl Zeiss LSM780 confocal microscope, and digitized images were generated using Carl Zeiss ZEN software (Zeiss, Jena, Germany).

### Statistical Analysis

All statistical analyses were performed using SPSS software (SPSS Inc., Chicago, IL, USA) where P ≤ 0.05 were considered significant. Roc curves were used to determine the optimal threshold for positive and negative values of galectin expression (plasma and cancer cells) as well as for the threshold for low and high CA125 plasma levels. Protein expression of galectins were correlated to one another using Pearson’s correlation test (two-tailed) and correlated to the patients’ characteristics using Fisher exact test, T test and Kruskal Wallis Test. Overall survival (OS) and disease free survival (DFS) were calculated as the time elapsed between the day of the diagnosis and the death of the patient or the clinically proven recurrence, respectively. Paclitaxel/Carboplatin resistance was considered as a recurrence of the disease in the 2 years following the last day of chemotherapy treatment. Survival curves (OS, DSF and Paclitaxel/Carboplatin resistance) were plotted by the Kaplan-Meier estimator and compared using the log rank test. Univariate and multivariate Cox proportional hazard models were used to determine the hazard ratio for each marker. The age of the patients at diagnosis, FIGO stage, residual disease and CA125 levels were included in the multivariate analysis.

## Electronic supplementary material

Supplementary info

## References

[1] Suh DH (2017). Major clinical research advances in gynecologic cancer in 2016: 10-year special edition. Journal of Gynecologic Oncology.

[2] Salomon-Perzynski A, Salomon-Perzynska M, Michalski B, Skrzypulec-Plinta V (2017). High-grade serous ovarian cancer: the clone wars. Archives of Gynecology and Obstetrics.

[3] Le Page C, Huntsman DG, Provencher DM, Mes-Masson AM (2010). Predictive and prognostic protein biomarkers in epithelial ovarian cancer: recommendation for future studies. Cancers.

[4] Felder M (2014). MUC16 (CA125): tumor biomarker to cancer therapy, a work in progress. Molecular Cancer.

[5] Mogensen O, Mogensen B, Jakobsen A (1990). Predictive value of CA 125 during early chemotherapy of advanced ovarian cancer. Gynecologic Oncology.

[6] Schwartz PE (1987). Circulating tumor markers in the monitoring of gynecologic malignancies. Cancer.

[7] Dimitroff CJ (2015). Galectin-Binding O-Glycosylations as Regulators of Malignancy. Cancer Research.

[8] Salatino M, Rabinovich GA (2011). Fine-tuning antitumor responses through the control of galectin-glycan interactions: an overview. Methods in Moecularl Biology.

[9] Jung EJ (2007). Galectin-1 expression in cancer-associated stromal cells correlates tumor invasiveness and tumor progression in breast cancer. International Journal of Cancer.

[10] van den Brule F (2003). Galectin-1 accumulation in the ovary carcinoma peritumoral stroma is induced by ovary carcinoma cells and affects both cancer cell proliferation and adhesion to laminin-1 and fibronectin. Laboratory Investigation.

[11] Wu MH (2011). Targeting galectin-1 in carcinoma-associated fibroblasts inhibits oral squamous cell carcinoma metastasis by downregulating MCP-1/CCL2 expression. Clinical Cancer Research.

[12] Rabinovich GA, Croci DO (2012). Regulatory circuits mediated by lectin-glycan interactions in autoimmunity and cancer. Immunity.

[13] Cagnoni AJ, Perez Saez JM, Rabinovich GA, Marino KV (2016). Turning-Off Signaling by Siglecs, Selectins, and Galectins: Chemical Inhibition of Glycan-Dependent Interactions in Cancer. Frontiers in Oncology.

[14] Grosset AA (2014). Cytosolic galectin-7 impairs p53 functions and induces chemoresistance in breast cancer cells. BMC Cancer.

[15] Liu FT, Rabinovich GA (2005). Galectins as modulators of tumour progression. Nature Reviews. Cancer.

[16] Su YC (2016). Galectin-1-Induced Autophagy Facilitates Cisplatin Resistance of Hepatocellular Carcinoma. PloS One.

[17] Dalotto-Moreno T (2013). Targeting galectin-1 overcomes breast cancer-associated immunosuppression and prevents metastatic disease. Cancer Research.

[18] Ito K (2012). Galectin-1 as a potent target for cancer therapy: role in the tumor microenvironment. Cancer Metastasis Reviews.

[19] Salatino M, Dalotto-Moreno T, Rabinovich GA (2013). Thwarting galectin-induced immunosuppression in breast cancer. Oncoimmunology.

[20] Liu B (2010). Ovarian cancer immunotherapy: opportunities, progresses and challenges. Journal of Hematology & Oncology.

[21] Mellman I, Coukos G, Dranoff G (2011). Cancer immunotherapy comes of age. Nature.

[22] von Mensdorff-Pouilly, S. in *Encyclopedia of Cancer* 2698–2704 (Springer Berlin Heidelberg, 2011).

[23] Compagno D, Laderach DJ, Gentilini L, Jaworski FM, Rabinovich GA (2013). Delineating the “galectin signature” of the tumor microenvironment. Oncoimmunology.

[24] Laderach DJ (2013). A unique galectin signature in human prostate cancer progression suggests galectin-1 as a key target for treatment of advanced disease. Cancer Research.

[25] Laderach DJ, Gentilini L, Jaworski FM, Compagno D (2013). Galectins as new prognostic markers and potential therapeutic targets for advanced prostate cancers. Prostate Cancer.

[26] Payton S (2012). Prostate cancer: ‘Galectin signature’ reveals gal-1 as key player in angiogenesis. Nature Reviews. Urology.

[27] Grosset, A. A. *et al*. Galectin signatures contribute to the heterogeneity of breast cancer and provide new prognostic information and therapeutic targets. *Oncotarget* (2016). 10.18632/oncotarget.7784 PMC4951281 26933916

[28] Kim HJ (2012). High galectin-1 expression correlates with poor prognosis and is involved in epithelial ovarian cancer proliferation and invasion. European Journal of Cancer.

[29] Zhang P (2014). *Galectin-1 overexpressi*on promotes progression and chemoresistance to cisplatin in epithelial ovarian cancer. Cell Death & Disease.

[30] Fredriksson S (2008). Multiplexed proximity ligation assays to profile putative plasma biomarkers relevant to pancreatic and ovarian cancer. Clinical Chemistry.

[31] Casbas-Hernandez P (2015). Tumor intrinsic subtype is reflected in cancer-adjacent tissue. Cancer Epidemiology, Biomarkers Prevention.

[32] Jezequel P (2015). Gene-expression molecular subtyping of triple-negative breast cancer tumours: importance of immune response. Breast Cancer Research.

[33] Sevenich L (2014). Analysis of tumour- and stroma-supplied proteolytic networks reveals a brain-metastasis-promoting role for cathepsin S. Nature Cell Biology.

[34] Winslow S, Leandersson K, Edsjo A, Larsson C (2015). Prognostic stromal gene signatures in breast cancer. Breast Cancer Research.

[35] Punt S (2015). Galectin-1, -3 and -9 Expression and Clinical Significance in Squamous Cervical Cancer. PloS One.

[36] Thijssen VL, Heusschen R, Caers J, Griffioen AW (2015). Galectin expression in cancer diagnosis and prognosis: A systematic review. Biochimica Biophysica Acta.

[37] Enninga EA, Nevala WK, Holtan SG, Leontovich AA, Markovic SN (2016). Galectin-9 modulates immunity by promoting Th2/M2 differentiation and impacts survival in patients with metastatic melanoma. Melanoma Research.

[38] Demers M, Magnaldo T, St-Pierre Y (2005). A novel function for galectin-7: promoting tumorigenesis by up-regulating MMP-9 gene expression. Cancer Research.

[39] Demers M (2010). Overexpression of galectin-7, a myoepithelial cell marker, enhances spontaneous metastasis of breast cancer cells. American Journal of Pathology.

[40] Ueda S, Kuwabara I, Liu FT (2004). Suppression of tumor growth by galectin-7 gene transfer. Cancer Research.

[41] Califice S, Castronovo V, Bracke M, van den Brule F (2004). Dual activities of galectin-3 in human prostate cancer: tumor suppression of nuclear galectin-3 vs tumor promotion of cytoplasmic galectin-3. Oncogene.

[42] St-Pierre Y, Campion CG, Grosset AA (2012). A distinctive role for galectin-7 in cancer?. Frontiers in Bioscience (Landmark Ed).

[43] Funasaka T, Raz A, Nangia-Makker P (2014). Nuclear transport of galectin-3 and its therapeutic implications. Seminars in Cancer Biology.

[44] Grosset AA (2016). Galectin signatures contribute to the heterogeneity of breast cancer and provide new prognostic information and therapeutic targets. Oncotarget.

[45] Colombo PE (2014). Sensitivity and resistance to treatment in the primary management of epithelial ovarian cancer. Critical Reviews in Oncology/Hematology.

[46] Cho HY, Kim K, Kim YB, Kim H, No JH (2017). Expression Patterns of Nrf2 and Keap1 in Ovarian Cancer Cells and their Prognostic Role in Disease Recurrence and Patient Survival. International Journal of Gynecological Cancer.

[47] Scalici JM (2017). Mesothelium expression of vascular cell adhesion molecule-1 (VCAM-1) is associated with an unfavorable prognosis in epithelial ovarian cancer (EOC). Cancer.

[48] Banh A (2011). Tumor galectin-1 mediates tumor growth and metastasis through regulation of T-cell apoptosis. Cancer Research.

[49] He J, Baum LG (2004). Presentation of galectin-1 by extracellular matrix triggers T cell death. The Journal of Biological Chemistry.

[50] Perillo NL, Pace KE, Seilhamer JJ, Baum LG (1995). Apoptosis of T cells mediated by galectin-1. Nature.

[51] Rubinstein N (2004). Targeted inhibition of galectin-1 gene expression in tumor cells results in heightened T cell-mediated rejection; A potential mechanism of tumor-immune privilege. Cancer Cell.

[52] Kamper P (2011). Proteomic analysis identifies galectin-1 as a predictive biomarker for relapsed/refractory disease in classical Hodgkin lymphoma. Blood.

[53] Ouyang J (2013). Galectin-1 serum levels reflect tumor burden and adverse clinical features in classical Hodgkin lymphoma. Blood.

[54] Saussez S (2007). High level of galectin-1 expression is a negative prognostic predictor of recurrence in laryngeal squamous cell carcinomas. International Journal of Oncology.

[55] Saussez S (2008). The determination of the levels of circulating galectin-1 and -3 in HNSCC patients could be used to monitor tumor progression and/or responses to therapy. Oral Oncology.

[56] Schulkens IA (2014). Galectin expression profiling identifies galectin-1 and Galectin-9Delta5 as prognostic factors in stage I/II non-small cell lung cancer. PloS One.

[57] Szoke T (2005). Prognostic significance of endogenous adhesion/growth-regulatory lectins in lung cancer. Oncology.

[58] Chen L (2015). Clinical implication of the serum galectin-1 expression in epithelial ovarian cancer patients. Journal of Ovarian Research.

[59] Chagan-Yasutan H (2013). Galectin-9 plasma levels reflect adverse hematological and immunological features in acute dengue virus infection. Journal of Clinical Virology.

[60] Mengshol JA (2010). A crucial role for Kupffer cell-derived galectin-9 in regulation of T cell immunity in hepatitis C infection. PloS One.

[61] Tandon R (2014). Galectin-9 is rapidly released during acute HIV-1 infection and remains sustained at high levels despite viral suppression even in elite controllers. AIDS Research and Human Retroviruses.

[62] Turcotte M (2015). CD73 is associated with poor prognosis in high-grade serous ovarian cancer. Cancer Research.

[63] Fleury H (2015). *Novel high-grade* serous epithelial ovarian cancer cell lines that reflect the molecular diversity of both the sporadic and hereditary disease. Genes & Cancer.

[64] Letourneau IJ (2012). Derivation and characterization of matched cell lines from primary and recurrent serous ovarian cancer. BMC Cancer.

