# Supplementary material for: Tissue and plasma levels of galectins in patients with high grade serous ovarian carcinoma as new predictive biomarkers
Source: Sci Rep. 2017 Oct 16;7:13244. doi: 10.1038/s41598-017-13802-5 (PMC5643335; doi:10.1038/s41598-017-13802-5)
Supplement: Supplementary file 1 — Supplementary info [file 41598_2017_13802_MOESM1_ESM.pdf]

Tissue and plasma levels of galectins in patients with high grade serous ovarian carcinoma as new predictive biomarkers

Marilyne Labrie[1], Lorenna Oliveira Fernandes De Araujo[1], Laudine Communal[2,3], Anne-Marie Mes-Masson[2,3,4] and Yves St-Pierre[1]

Supplementary File

Supplementary Table I

Supplementary table I: Patients characteristics (multi-subtype tissue microarray (n=63))

	**Clear cell (n=12)**	**Endometrioid (n=21)**	**Mucinous (n=14)**	**Serous (n=16)**
**Age range (mean)**	47-73 (57)	43-80 (59)	28-82 (54)	33-79 (57)
**Grade**				
**Borderline**	0	0	12	3
**I (Low)**	0	11	1	2
**II**	4	5	1	5
**III (High)**	8	5	0	6
**Stage**				
**I**	6	13	11	2
**II**	2	1	1	3
**III**	4	6	2	10
**IV**	0	1	0	1

Supplementary Table II

Supplementary table II: Patients characteristics (HGSC tissue microarray (n=209))

**Age range (median)**	36-89 (64.5)
**Figo Stage**	
I	13
II	23
III	148
IV	25
**CA-125 range (median)**	5-13763 (667)
**Residual disease**	
No residual disease	40
≤ 1 cm	51
>1 cm ≤ 2 cm	30
> 2 cm	50
Unknown	38
**Chemotherapy**	
Paclitaxel/carboplatin	147
Other	60
Unknown	2
**Reccurence**	
Yes	172
No	37
**Mortality**	
Yes	140
No	69

Supplementary Table III

Supplementary Table III: Correlation between the patients characteristics and galectins expression in cancer cells and stroma of HGSC.

	Gal-1		Gal-3		Gal-7		Gal-8		Gal-9	
	**Low**	**High**	**Low**	**High**	**Low**	**High**	**Low**	**High**	**Low**	**High**
Cancer cells										
**Age**										
Mean	61	62	64	59	59	63	63	61	62	61
**Figo Stage**	***P = 0.056***									
Low (I-II)	29 (14)	13 (6)	13 (7)	20 (11)	13 (7)	20 (11)	17 (9)	17 (9)	16 (8)	18 (9)
High (III-IV)	84 (41)	**77 (38)**	58 (31)	98 (52)	71 (38)	85 (45)	67 (34)	98 (49)	82 (43)	74 (39)
**CA-125**	***P = 0.016***									
Median	453.36	**809.5**	587.9	697.44	765.5	559.5	530.32	618	553	697.44
**Residual disease**										
No	21 (13)	15 (9)	12 (8)	25 (16)	17 (11)	20 (13)	18 (11)	20 (12)	21 (13)	16 (10)
Yes	63 (40)	59 (37)	50 (32)	69 (44)	51 (33)	68 (44)	58 (35)	70 (42)	60 (38)	60 (38)
**Recurrence**	***P = 0.092***						***P = 0.065***			
No	23 (12)	12 (6)	15 (8)	20 (11)	18 (10)	17 (9)	21 (11)	16 (8)	21 (11)	14 (8)
Yes	75 (40)	**78 (41)**	56 (30)	94 (51)	63 (34)	87 (47)	62 (32)	**96 (49)**	76 (41)	75 (40)
**Death**										
No	49 (28)	36 (21)	30 (16)	56 (30)	41 (22)	45 (24)	36 (18)	53 (27)	47 (25)	38 (20)
Yes	53 (31)	34 (20)	41 (22)	62 (33)	43 (23)	60 (32)	48 (24)	62 (31)	51 (27)	54 (28)
Stroma										
**Age**										
Mean	61	61	60	62	61	1	61	61	61	62
**Figo Stage**										
Low (I-II)	13 (7)	19 (10)	16 (9)	16 (9)	31 (17)	1 (1)	22 (12)	11 (6)	10 (5)	23 (13)
High (III-IV)	49 (26)	111 (58)	92 (49)	63 (34)	141 (75)	14 (7)	109 (58)	47 (25)	51 (28)	100 (54)
**CA-125**	***P = 0.125***								***P = 0.074***	
Median	453.4	793	548.5	828.5	589.5	769.5	618	587.9	510	**767.5**
**Residual desease**										
No	10 (6)	26 (16)	20 (13)	17 (11)	35 (23)	2 (1)	23 (15)	15 (9)	13 (9)	23 (15)
Yes	42 (27)	80 (51)	72 (47)	45 (29)	107 (69)	10 (6)	85 (54)	35 (22)	34 (23)	81(54)
**Recurrence**	***P = 0.008***									
No	18 (10)	17 (6)	19 (10)	16 (9)	31 (17)	4 (2)	24 (13)	11 (6)	11 (6)	23 (13)
Yes	41 (22)	112 **(60)**	86 (47)	62 (34)	137 (75)	11 (6)	103 (56)	47 (25)	46 (26)	100 (56)
**Death**							***P = 0.027***			
No	31 (16)	54 (28)	46 (25)	38 (20)	77 (41)	7 (4)	53 (28)	**78 (41)**	24 (13)	58 (32)
Yes	31 (16)	76 (40)	62 (33)	41 (22)	95 (51)	8 (4)	34 (18)	24 (13)	37 (20)	65 (35)

Supplementary Table IV

Supplementary table IV: Correlation between the presence of galectins in the tumor and the plasma.

			Epithelium		Stroma	
			Negative	Positive	Negative	Positive
Galectin-8			P = 0.280		P = 0.007	
ELISA	Negative	n	28	54	46	33
		%	34.1	65.9	58.2	41.8
	Positive	n	24	30	41	9
		%	44.4	55.6	82	18
Galectin-9			P = 0.152		P = 0.689	
ELISA	Negative	n	34	21	17	36
		%	61.8	38.2	32.1	67.9
	Positive	n	35	38	19	50
		%	47.9	52.1	27.5	72.5

Supplementary figure 1

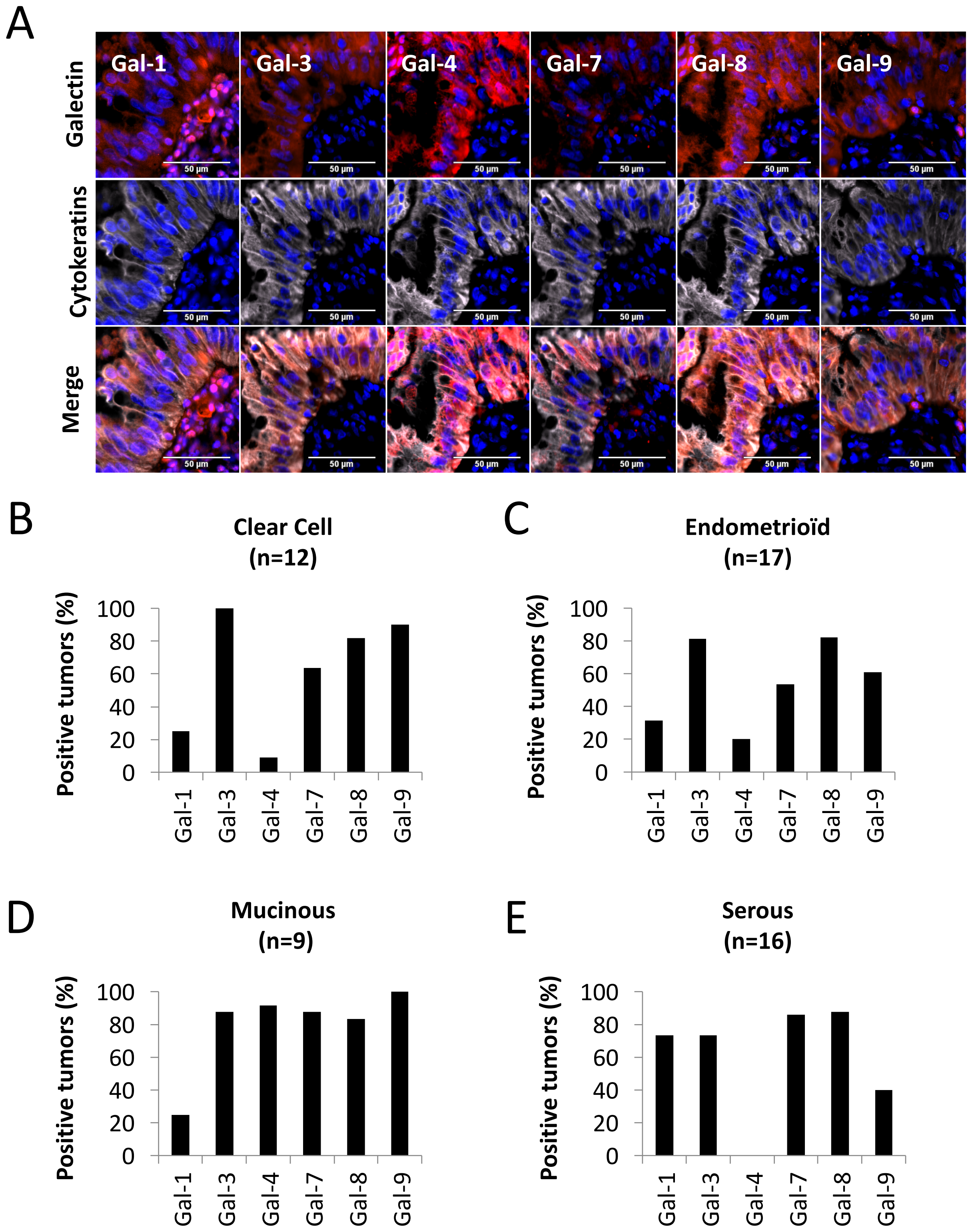

Supplementary figure 1: Galectins expression in different histological subtypes of ovarian cancer. (A) Positive IHF staining of gal-1, -3, -4, -7 -8 and -9 in a single mucinous tumor. Cytokeratin staining was used to identify epithelial cancer cells. Bar represent 50 µm. Percentage of positive tumors displaying expression of gal-1, -3, -4, -7, -8 and -9 in (B) clear cell, (C) endometrioid, (D) mucinous and (E) serous ovarian tumors.

Supplementary figure 2

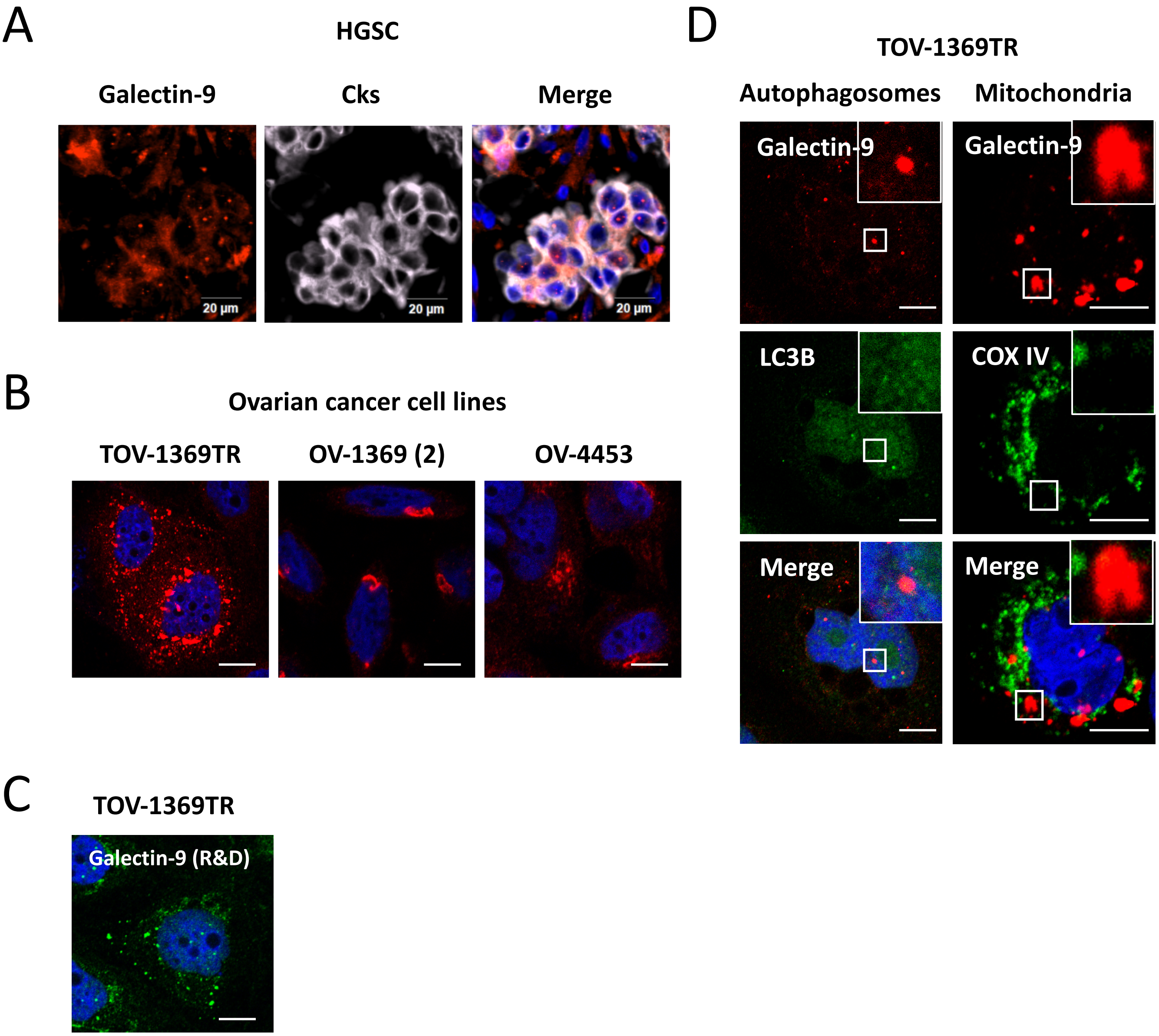

Supplementary figure 2: Gal-9P in ovarian cancer cells. (A) IHF staining of gal-9P in HGSC. Cytokeratin staining was used to identify epithelial cancer cells. Bar represent 20 µm. (B) IF staining of gal-9 (red) was detected by confocal imagery in Tov-1369TR, Tov-1369(2) and OV-4453 ovarian cancer cell lines. Nuclei were stained with DAPI (blue). (C) IF staining of gal-9 (red) was detected by confocal imagery in Tov-1369TR with a goat anti-gal-9 antibody (R&D). (D) IF staining of gal-9 (red), LC3B (green, left panel) and COX IV (green, right panel) in TOV-1369TR cells. LC3B and Cox IV antibodies were used to stain autophagosome and mitochondria, respectively. Nuclei were stained with DAPI (blue). Bars represent 10 µm.

Supplementary figure 3

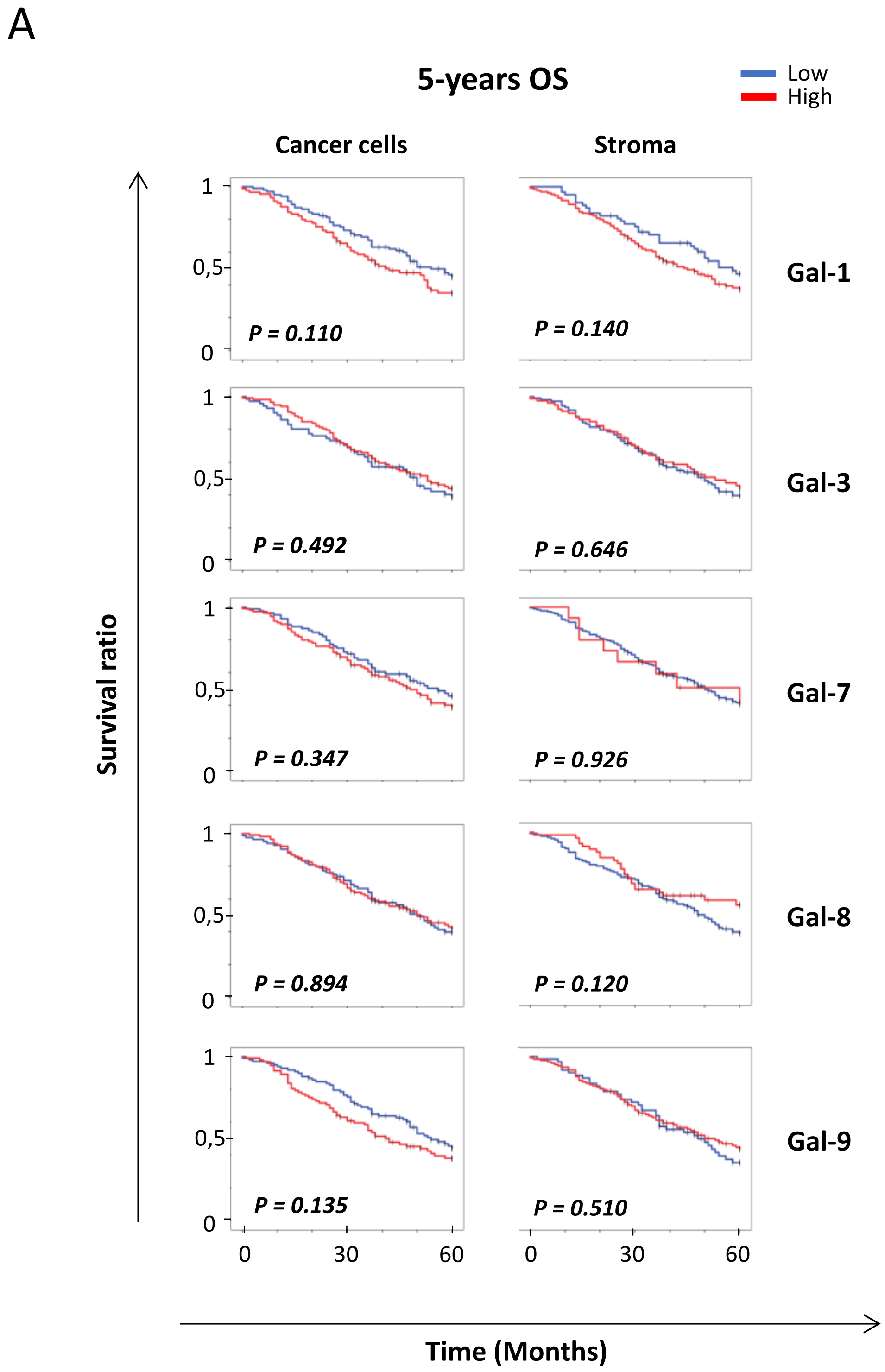

Supplementary figure 3

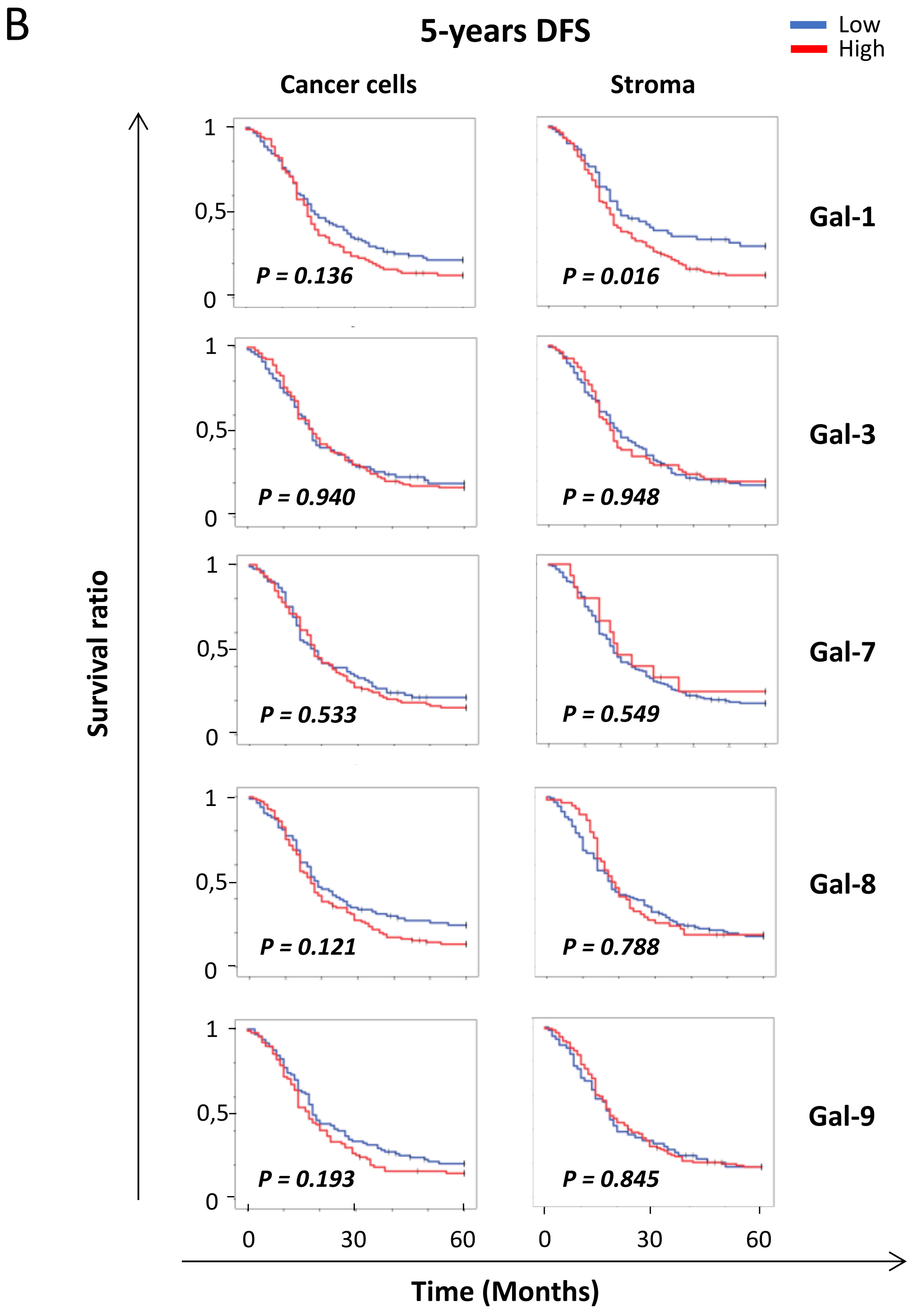

Supplementary figure 3

C

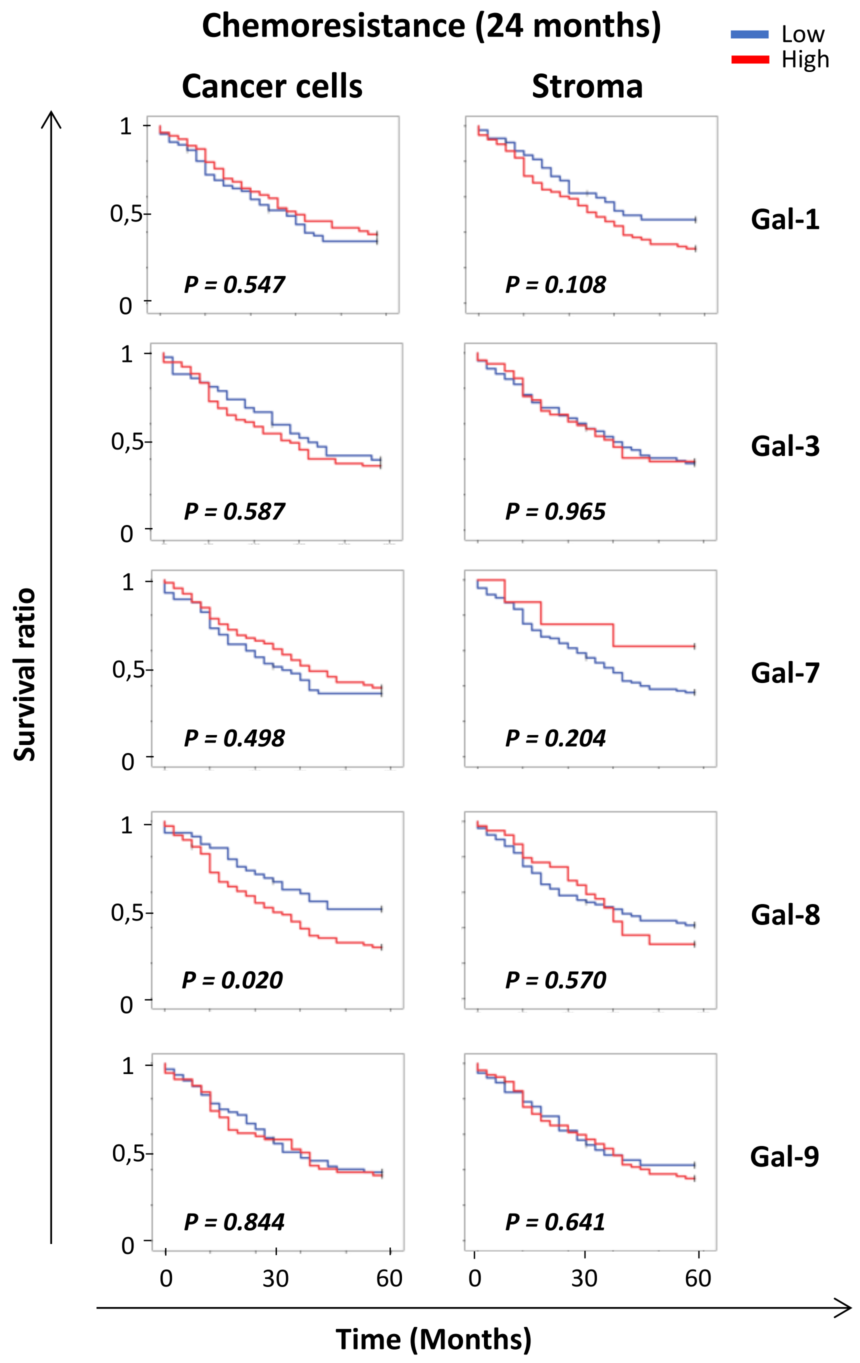

Supplementary figure 3

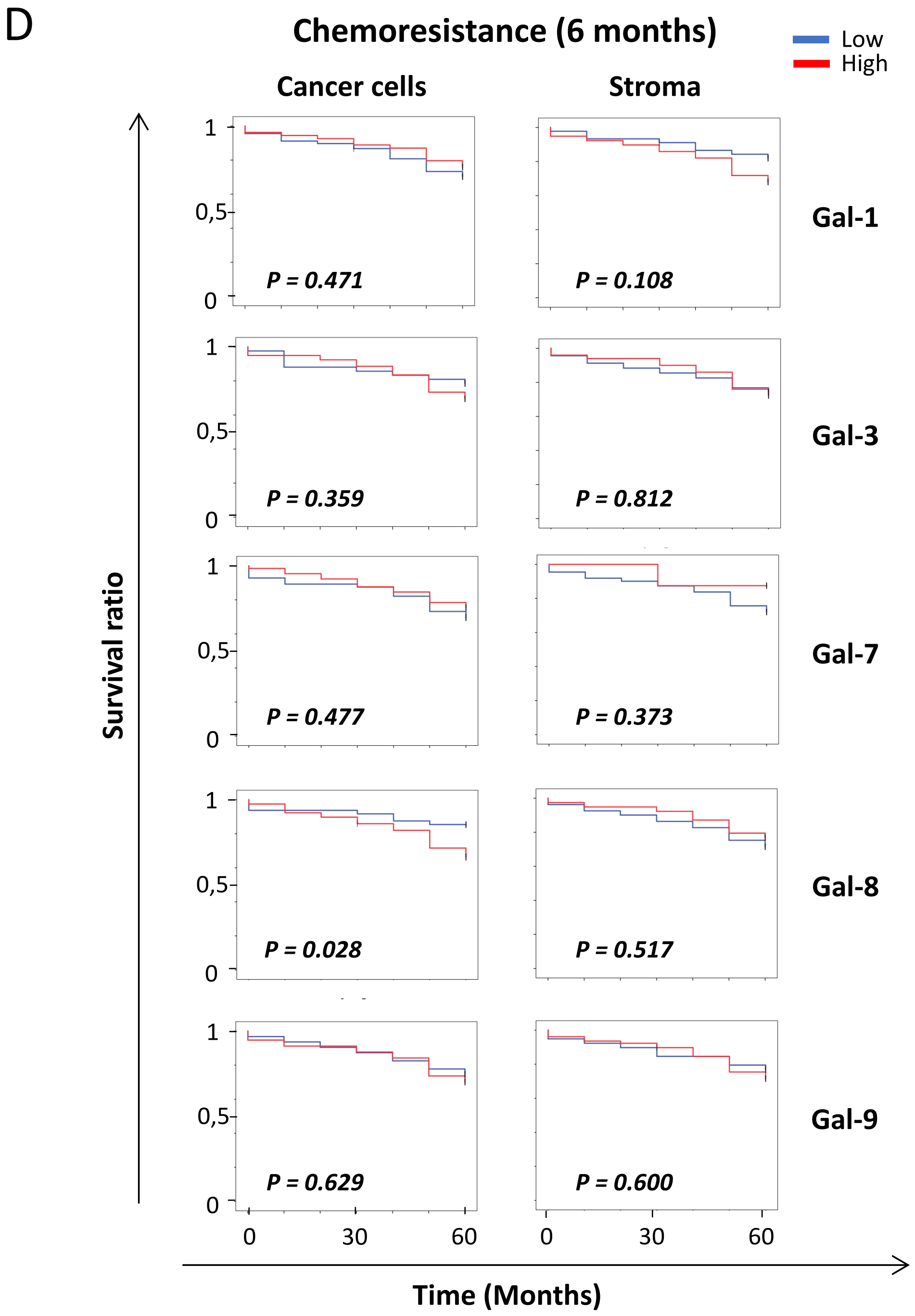

Supplementary figure 3: Galectins predictive value of OS, DFS and chemoresistance. Kaplan-Meier curves of (A) 5-years OS, (B) 5-years DFS and chemoresistance analyzed at (C) 24 months and (D) 6 months post-treatment according to epithelial or stromal presence of gal-1, -3, -7, -8 and -9. Blue bar: galectin negative tumors, red bar: galectin positive tumors.

Supplementary figure 4

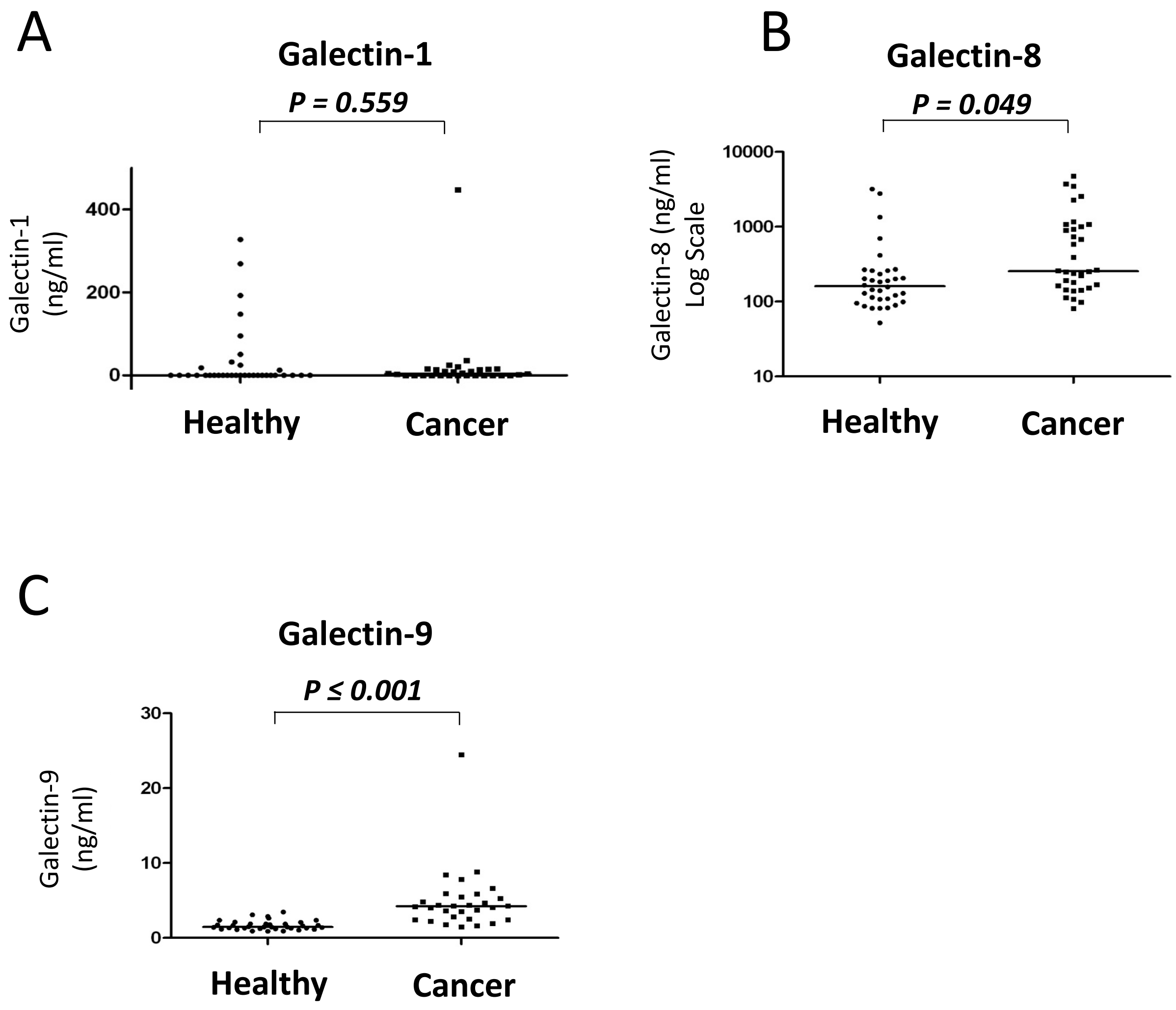

Supplementary figure 4: Galectins concentration in the plasma of healthy donors and ovarian cancer patients. Levels of circulating gal-1, gal-8 and gal-9 in 35 healthy donors and 35 cancer patients.
